# Supplementary material for: Multi‐dataset identification of innovative feature genes and molecular mechanisms in keratoconus
Source: J Cell Mol Med. 2024 Sep 19;28(18):e70079. doi: 10.1111/jcmm.70079 (PMC11412914; doi:10.1111/jcmm.70079)
Supplement: Supplementary file 2 — Material S2. [file JCMM-28-e70079-s002.zip › 7.GSEA analysis/3.DBNDD1/KEGG_BUTANOATE_METABOLISM.html]

Details for gene set KEGG\_BUTANOATE\_METABOLISM[GSEA]

|  || Dataset | DBNDD1.group.cls#h\_versus\_l |
| Phenotype | group.cls#h\_versus\_l |
| Upregulated in class | h |
| GeneSet | KEGG\_BUTANOATE\_METABOLISM |
| Enrichment Score (ES) | 0.24192435 |
| Normalized Enrichment Score (NES) | 0.7064625 |
| Nominal p-value | 0.8398357 |
| FDR q-value | 1.0 |
| FWER p-Value | 1.0 |
Table: GSEA Results Summary

  

Fig 1: Enrichment plot: KEGG\_BUTANOATE\_METABOLISM      
 Profile of the Running ES Score & Positions of GeneSet Members on the Rank Ordered List

  

| SYMBOL | TITLE | RANK IN GENE LIST | RANK METRIC SCORE | RUNNING ES | CORE ENRICHMENT || 1 | HMGCS2 | na | 275 | 0.517 | 0.1339 | Yes |
| 2 | ACSM1 | na | 606 | 0.428 | 0.2419 | Yes |
| 3 | GAD2 | na | 5927 | 0.152 | 0.1304 | No |
| 4 | ACSM4 | na | 7084 | 0.122 | 0.1307 | No |
| 5 | HMGCL | na | 7626 | 0.111 | 0.1457 | No |
| 6 | ALDH5A1 | na | 9582 | 0.077 | 0.1104 | No |
| 7 | AACS | na | 11162 | 0.056 | 0.0804 | No |
| 8 | ABAT | na | 12350 | 0.044 | 0.0582 | No |
| 9 | ACSM5 | na | 12552 | 0.042 | 0.0640 | No |
| 10 | ALDH3A2 | na | 12835 | 0.040 | 0.0667 | No |
| 11 | L2HGDH | na | 13904 | 0.031 | 0.0444 | No |
| 12 | BDH1 | na | 14227 | 0.028 | 0.0428 | No |
| 13 | ECHS1 | na | 14374 | 0.027 | 0.0461 | No |
| 14 | ACAT1 | na | 14381 | 0.027 | 0.0535 | No |
| 15 | ACAT2 | na | 15183 | 0.021 | 0.0363 | No |
| 16 | ACSM2A | na | 15392 | 0.020 | 0.0358 | No |
| 17 | PDHA2 | na | 15714 | 0.018 | 0.0314 | No |
| 18 | OXCT1 | na | 15786 | 0.017 | 0.0341 | No |
| 19 | HMGCS1 | na | 16203 | 0.014 | 0.0261 | No |
| 20 | PDHB | na | 16264 | 0.014 | 0.0282 | No |
| 21 | HADHA | na | 17010 | 0.009 | 0.0093 | No |
| 22 | HADH | na | 17746 | 0.005 | -0.0106 | No |
| 23 | ALDH7A1 | na | 18637 | -0.002 | -0.0358 | No |
| 24 | ALDH1B1 | na | 22410 | -0.036 | -0.1345 | No |
| 25 | ALDH9A1 | na | 22786 | -0.041 | -0.1342 | No |
| 26 | ACADS | na | 23379 | -0.048 | -0.1381 | No |
| 27 | PDHA1 | na | 24150 | -0.058 | -0.1443 | No |
| 28 | GAD1 | na | 25028 | -0.071 | -0.1501 | No |
| 29 | ACSM3 | na | 28060 | -0.133 | -0.2011 | No |
| 30 | BDH2 | na | 29050 | -0.158 | -0.1861 | No |
| 31 | EHHADH | na | 30010 | -0.189 | -0.1619 | No |
| 32 | OXCT2 | na | 31743 | -0.251 | -0.1430 | No |
| 33 | ALDH2 | na | 33235 | -0.347 | -0.0907 | No |
| 34 | AKR1B10 | na | 34307 | -0.487 | 0.0121 | No |
Table: GSEA details [plain text format]

  

Fig 2: KEGG\_BUTANOATE\_METABOLISM      
 Blue-Pink O' Gram in the Space of the Analyzed GeneSet

  

Fig 3: KEGG\_BUTANOATE\_METABOLISM: Random ES distribution      
 Gene set null distribution of ES for **KEGG\_BUTANOATE\_METABOLISM**
